# Comparing the findings and diagnostic sensitivity of cardiovascular magnetic resonance in biopsy confirmed acute myocarditis with infarct-like vs. heart failure presentation

**DOI:** 10.1186/s12968-022-00903-y

**Published:** 2022-12-07

**Authors:** Karim Hassan, Anton Doubell, Charles Kyriakakis, Lloyd Joubert, Pieter-Paul Robbertse, Gert Van Zyl, Dan Zaharie, Philip Herbst

**Affiliations:** 1 Division of Cardiology, Department of Medicine, Stellenbosch University, Tygerberg Hospital, E8, 8/F Tygerberg Hospital, Francie Van Zijl Drive, Parow, Cape Town, 7505 South Africa; 2 Division of Medical Virology, National Health Laboratory Services, Tygerberg Hospital, Cape Town, South Africa; 3 Division of Anatomical Pathology, National Health Laboratory Services, Tygerberg Hospital, Cape Town, South Africa

**Keywords:** Myocarditis, Viral myocarditis, Cardiac magnetic resonance imaging, Parametric mapping, Lake Louise criteria

## Abstract

**Background:**

Cardiovascular magnetic resonance (CMR) is considered the reference imaging modality in providing a non-invasive diagnosis of acute myocarditis (AM), as it allows for the detection of myocardial injury associated with AM. However, the diagnostic sensitivity and pattern of CMR findings appear to differ according to clinical presentation.

**Methods:**

This is a retrospective cross-sectional study. Consecutive adult patients presenting to a single tertiary centre in South Africa between August 2017 and January 2022 with AM confirmed on endomyocardial biopsy (EMB) were enrolled. Patients with infarct-like symptoms, defined as those presenting primarily with chest pain syndrome with associated ST-T wave changes on electrocardiogram, or heart failure (HF) symptoms, defined as clinical signs and symptoms of HF without significant chest discomfort, were compared using contrasted CMR and parametric techniques with EMB confirmation of AM as diagnostic gold standard.

**Results:**

Forty-one patients were identified including 23 (56%) with infarct-like symptoms and 18 (44%) with HF symptoms. On CMR, the infarct-like group had significantly higher ejection fractions of both ventricles (LVEF 55.3 ± 15.3% vs. 34.4 ± 13.5%, p < 0.001; RVEF 57.3 ± 10.9% vs. 42.9 ± 18.2%, p = 0.008), without significant differences in end diastolic volumes (LVEDVI 82.7 ± 30.3 ml/m^2^ vs. 103.4 ± 35.9 ml/m^2^, p = 0.06; RVEDVI 73.7 ± 22.1 ml/m^2^ vs. 83.9 ± 29.9 ml/m^2^, p = 0.25). Myocardial oedema was detected more frequently on T2-weighted imaging (91.3% vs. 61.1%, p = 0.03) and in more myocardial segments [3.0 (IQR 2.0–4.0) vs. 1.0 (IQR 0–1.0), p = 0.003] in the infarct-like group. Despite the absence of a significant statistical difference in the prevalence of late gadolinium enhancement (LGE) between the two groups (95.7% vs. 72.2%, p = 0.07), the infarct-like group had LGE detectable in significantly more ventricular segments [4.5 (IQR 2.3–6.0) vs. 2.0 (IQR 0–3.3), p = 0.02] and in a different distribution. The sensitivity of the original Lake Louise Criteria (LLC) was 91.3% in infarct-like patients and 55.6% in HF patients. When the updated LLC, which included the use of parametric myocardial mapping techniques, were applied, the sensitivity improved to 95.7% and 72.2% respectively.

**Conclusion:**

The pattern of CMR findings and its diagnostic sensitivity appears to differ in AM patients presenting with infarct-like and HF symptoms. Although the sensitivity of the LLC improved with the addition of parametric mapping in the HF group, it remained lower than that of the infarct-like group, and suggests that EMB should be considered earlier in the course of patients with clinically suspected AM presenting with HF.

**Supplementary Information:**

The online version contains supplementary material available at 10.1186/s12968-022-00903-y.

## Background

Myocarditis is defined as an inflammatory disease of the heart muscle, diagnosed by established histological, immunological and immunohistochemical (IHC) criteria [1]. Its clinical diagnosis is often challenging due to the heterogeneity in clinical presentations [1, 2]. Diagnostic difficulty is further compounded by the lack of pathognomonic findings of acute myocarditis (AM) on bedside and laboratory investigations, and transthoracic echocardiography (TTE).

Although endomyocardial biopsy (EMB) remains the gold standard for the diagnosis of AM [1, 2] and despite safety data, even in low volume centres [3], it is infrequently sought in patients with clinically suspected AM due to its invasive nature and the perceived low diagnostic yield resulting from sampling error. As a result, cardiovascular magnetic resonance (CMR) is now considered the reference imaging modality for providing a non-invasive diagnosis of AM [4–7].

CMR allows for an accurate morphological and functional cardiac assessment and importantly, adds the ability to non-invasively characterise myocardial tissue, which sets it apart from TTE for the diagnosis of myocarditis. The Lake Louise Criteria (LLC), originally published in 2009, was the standard for CMR diagnosis of AM and takes into account the three markers of myocardial injury, namely, intracellular and interstitial oedema on T2-weighted imaging, hyperaemia and capillary leakage with early gadolinium enhancement (EGE), and necrosis and fibrosis with late gadolinium enhancement (LGE) [6]. Its specificity and positive predictive value had been reported to be as high as 91% when two out of three markers of myocardial injury are present [6, 8, 9]. However, the sensitivity appeared somewhat lower and depended on the manner of presentation, ranging from 80% in patients with infarct-like presentation to 57% in patients presenting with heart failure (HF) [10].

The LLC were updated in 2018 with the incorporation of the assessment of both native T1 and T2 relaxation times using parametric myocardial mapping techniques, and required the fulfilment of at least one T2-based imaging criterion for oedema and at least one T1-based tissue characterisation criterion [7]. These changes have significantly improved both the specificity and diagnostic accuracy of the LLC, especially in patients who do not present with infarct-like symptoms [7, 11].

Due to limited access to CMR services in the developing world, the majority of studies investigating the CMR findings of patients with AM are conducted in the developed world. The CMR characteristics of African and South African patients with AM are currently unknown. Furthermore, the diagnosis of AM in the majority of these studies were based on clinical criteria, with only a minority of patients having undergone EMB.

In this retrospective study, we sought to compare the CMR findings of patients with confirmed AM on EMB who presented with either infarct-like or HF symptoms to a single tertiary centre in Cape Town, South Africa.

## Methods

### Population and study design

This is a single-centre retrospective cross-sectional study. Consecutive patients over the age of 18 years presenting to Tygerberg Hospital, Cape Town, South Africa between August 2017 and January 2022 who fulfilled the European Society of Cardiology’s (ESC) diagnostic criteria for clinically suspected AM [1] and had undergone all investigations as recommended by the ESC position paper on AM, including CMR and EMB, were screened for recruitment. Those presenting with infarct-like symptoms or symptoms of HF who fulfilled the histological or immunohistochemical criteria [12, 13] for the diagnosis of AM on EMB were included for analysis. All cases deemed to be related to coronavirus disease of 2019 (COVID-19) and its vaccinations were excluded. Written informed consent was obtained from all participants prior to CMR for participation in the cohort/registry.

AM was clinically suspected if patients presented with symptoms compatible with AM accompanied by at least one additional investigation supporting the diagnosis of AM. All patients underwent a full clinical evaluation. Routine laboratory studies including a full blood count, renal function, high sensitivity cardiac troponin T (hs-cTnT) and C-reactive protein (CRP) were performed. Additional laboratory studies were requested at the discretion of the attending physician. All patients also underwent a standard 12-lead electrocardiogram (ECG) and TTE as per standardised protocol described below. Coronary angiography was performed to exclude any significant epicardial coronary artery disease, defined as ≥ 50% stenosis in a single coronary artery segment. Both CMR and right ventricular EMB were performed on all patients as per the standard of care for patients with clinically suspected AM at our centre. The order in which CMR and EMB were performed depended on the availability at the time of admission.

Patients were categorised and compared according to the manner of their clinical presentation as previously described [1, 10]. An infarct-like presentation was defined as those presenting primarily with chest pain and ECG ST-T wave changes or without an increase in hs-cTnT and absence of angiographic evidence of significant epicardial coronary artery disease or recent plaque rupture. HF presentation was defined clinically based on presenting with symptoms and signs of HF, which included dyspnoea, orthopnoea, paroxysmal nocturnal dyspnoea elevated jugular venous pressure and peripheral oedema, as well as imaging evidence of pulmonary congestion on chest radiography. Chest discomfort, if present, was not a dominant feature of the clinical presentation. ECG changes, if present, were non-specific.

### Transthoracic echocardiography (TTE)

Comprehensive functional and structural 2-dimensional TTE were performed on all patients with Vivid S7 or Vivid E95 (General Electric Healthcare, Chicago, Illinois, USA). Measurements were performed in accordance with the British Society of Echocardiography guidelines [14].

### Cardiovascular magnetic resonance (CMR)

CMR was performed in accordance with recommendations as set out in the *Journal of the American College of Cardiology*’s white paper on CMR in myocarditis and 2018 update of CMR criteria for myocardial inflammation, as well as *the Journal of Cardiovascular Magnetic Resonanc*e’s 2013 and 2020 CMR protocol update [6, 7, 15, 16]. All imaging was done at the Tygerberg Hospital using a 1.5 T CMR system (Magnetom Avanto; Siemens Healthineers GmbH, Erlangen, Germany). CMR analysis was carried out using commercially available software (cvi^42^, Circle Cardiovascular Imaging, Alberta, Calgary, Canada).

Standard long axis- and a short axis stack of breath-held, retrospectively gated, balanced steady-state free precession (bSSFP) cine images were obtained. Endocardial and epicardial left ventricular (LV) borders were traced in short axis at end-diastole and end-systole to determine LV volume, mass and functional parameters. Papillary muscles were excluded from the blood pool. Regional wall motion abnormality (RWMA) was assessed qualitatively in all LV segments and deemed present if the specific segment was classified as ‘hypokinetic’, ‘akinetic’ or ‘dyskinetic’. Quantitative analysis of short tau inversion recovery (STIR) images was performed following region of interest (ROI) contouring in short axis at basal, mid and apical LV level. A skeletal muscle (serratus anterior) ROI was manually drawn in the same slice for calculation of myocardial to skeletal muscle signal intensity ratio (SIR). A SIR ≥ 2.0 was considered abnormal. Pre-contrast native T1 mapping images were obtained using a shortened modified Look-Locker inversion (ShMOLLI) sequence. Native T1 time of more than 1050 ms was considered abnormal [11, 17]. Standard T2-mapping was performed using a bSSFP readout sequence preceded by a multinomial T2-preparation module (Siemens Aera 1.5 T). T2 time exceeding 50 ms was considered abnormal, as this was two standard deviations above a value determined to be normal for our scanner in a control cohort (46 ± 2 ms) [11, 18]. EGE and LGE images were obtained with a T1-weighted, segmented, inversion recovery sequence performed at least 10 min after contrast administration. We used a standardised 17-segment model of the LV as outlined by the American Heart Association for regional assessment and to describe abnormalities [19]. A gadolinium based contrast agent (Gadovist^©^, Bayer Healthcare, Berlin, Germany) was administered at a standard cardiac dose of 0.2 ml/kg.

### CMR analysis

All post-processing and image analysis was carried out using commercially available software (cvi^42^, Circle Cardiovascular Imaging) by two experienced observers who were blinded to EMB results.

### Endomyocardial biopsy

Right ventricular (RV) septal biopsies were performed on all patients as described in detail previously [3]. At least six specimens were taken from different sections of the septum to improve sensitivity. Three to four specimens were fixed in 4% buffered formalin for histological and immunohistochemical analysis, while the remaining samples were transported in 0.9% saline for viral genome detection by polymerase chain reaction (PCR).

### Histopathological, immunohistochemical analysis and detection of viral genomes

Specimens were assessed by a single anatomical pathologist at the National Health Laboratory Services (NHLS). Light microscopy was performed on haemotoxylin and eosin stained slides, along with immunohistochemical testing using anti-CD3 (T lymphocytes), anti-CD163 (macrophages) and anti-HLA-DR to define the types of immune cells. Additional stains were performed at the discretion of the pathologist. AM was diagnosed by either the Dallas histological criteria or the World Health Organisation (WHO)/International Society and Federation of Cardiology (ISFC) immunohistochemical criteria [12, 13]. PCR was performed for a standard panel of myocarditis related viruses, which includes parvovirus B19 (PVB19), Epstein-Barr virus (EBV), human herpes virus (HHV) 1 and 2, cytomegalovirus (CMV), HHV-6, human adenoviruses, influenza A and B, and human enteroviruses (which include Coxsackie viruses).

### Statistical analysis

Statistical analysis was performed using SPSS (version 27.0, Statistical Package for the Social Sciences, International Business Machines, Inc., Armonk, New York, USA). Normality of data was determined using the Kolmogorov–Smirnov test. Continuous variables were expressed as absolute numbers with associated percentages, mean and standard deviation if normally distributed, or median and interquartile range if not normally distributed. Categorial variables were expressed as absolute numbers and percentages. Comparisons between groups were done by the use of Kruskal–Wallis or Mann–Whitney U test for non-normally distributed continuous variables and Student t test for normally distributed variables. The Chi-square and Fisher exact test were used for comparison of categorical variables. A 2-tailed *p* value < 0.05 was considered statistically significant.

## Results

Between August 2017 and January 2022, 41 patients with confirmed AM on EMB presented with either infarct-like (n = 23/56.1%) or HF (n = 18/43.9%) symptoms. The mean age of the two groups was similar (p = 0.78). The baseline demographics, laboratory investigations, TTE and EMB findings are summarised and compared in Table 1. Patients with infarct-like presentation had significantly higher median hs-cTnT (724 vs. 104, p = 0.002) and were more likely to have a hs-cTnT above the 99th centile at baseline (91.3% vs. 44.4%, p = 0.002). The prevalence of PVB19 on EMB was similar between the two groups (p = 0.53).

**Table 1:** Baseline characteristics of patients with endomyocardial biopsy confirmed acute myocarditis (n = 41)

	Infarct-like (n = 23)	Heart failure (n = 18)	p values
Demographics			
Age (years)	39.6 ± 13.9	38.3 ± 13.2	0.78
Sex, male (n, %)	16 (69.6)	8 (44.4)	0.13
HIV + (n, %)	4 (17.4)	6 (33.3)	0.29
Laboratory investigation			
Hs-cTnT (ng/L)	724 (270–1361)	104 (35–499)	0.002
Hs-cTnT > 100 ng/L (n, %)	21 (91.3)	8 (44.4)	0.002
TTE parameter			
LVEDD (mm)	48.1 ± 6.9	52.1 ± 6.1	0.06
Endomyocardial biopsy			
Dallas criteria (n, %)	8 (34.8)	6 (33.3)	1.00
IHC criteria (n, %)	15 (65.2)	12 (66.7)	
Viral genomes detected (n, %)	17 (73.9)	11 (61.1)	0.50
Parvovirus B19	12	7	0.53
Epstein-Barr Virus	2	2	
Human Herpes Virus 6	1	1	
Human Bocavirus	1	0	
Enterovirus	0	0	
Adenovirus	0	0	
PVB19/EBV	1	0	
PVB19/EBV/HHV6	0	1	

*HIV* human immunodeficiency virus, *hsTnT* high sensitivity troponin T, *TTE* transthoracic echocardiography, *LVEDD* left ventricular end diastolic diameter, *PVB19* Parvovirus B19, *EBV* Epstein-Barr Virus, *HHV6* human herpesvirus 6

### CMR

The mean days from symptoms onset to CMR in the infarct-like group was 10.3 ± 8.8 days and was 15.6 ± 8.3 days for the HF group (p = 0.09).

### Morphology and function

The baseline findings of the LV and RV structural and functional assessments are summarised and compared in Table 2. Patients with infarct-like presentation had significantly higher LV ejection fraction (LVEF; (55.3% vs. 34.4%, p ≤ 0.001) and RV ejection fraction (RVEF; 57.3% vs. 42.9%, p = 0.008) and significantly lower indexed LV end-systolic volume (LVESV; 40.7 ml/m^2^ vs. 70.5 ml/m^2^, p = 0.008) and RV end systolic volumes (RVESV; 33.0 ml/m^2^ vs. 51.1 ml/m^2^, p = 0.03) when compared to patients with HF presentation. Those presenting with HF had significantly higher median segments of RWMA (2.0 vs. 14.0, p = 0.002).

**Table 2:** Cardiovascular magnetic resonance imaging findings (n = 41)

	Infarct-like (n = 23)	Heart failure (n = 18)	p values
LVEF (%)	55.3 ± 15.3	34.4 ± 13.5	< 0.001
LVEDVI (ml/m^2^)	82.7 ± 30.3	103.4 ± 35.9	0.06
LVESVI (ml/m^2^)	40.7 ± 30.1	70.5 ± 36.4	0.008
LV mass indexed (g/m^2^)	78.6 ± 29.0	89.4 ± 33.5	0.31
Max LV thickness (mm)	12.2 ± 3.5	11.2 ± 2.8	0.35
RWMA (segments)	2.0 (IQR 0–3.0)	14.0 (IQR 3.8–16.0)	0.002
RVEF (%)	57.3 ± 10.9	42.9 ± 18.2	0.008
RVEDVI (ml/m^2^)	73.7 ± 22.1	83.9 ± 29.9	0.25
RVESVI (ml/m^2^)	33.0 ± 16.4	51.1 ± 29.6	0.03
Pericardial effusion (n, %)	16 (69.6)	11 (61.1)	0.75
T2-weighted			
STIR			
Positive (n, %)	21 (91.3)	11 (61.1)	0.03
Total Segments (n)	3.0 (IQR 2.0–4.0)	1.0 (IQR 0–1.0)	0.003
Signal intensity ratio	2.3 ± 0.5	2.6 ± 0.7	0.39
T1-weighted			
EGE			
Positive (n, %)	17 (73.9)	10 (55.6)	0.32
Signal intensity ratio	8.1 (IQR 5.5–9.4)	4.1 (IQR 3.4–6.6)	0.06
LGE			
Positive (n, %)	22 (95.7)	13 (72.2)	0.07
Total segments (n)	4.5 (IQR 2.3–6.0)	2.0 (IQR 0–3.3)	0.02
Pattern			
Inferolateral (n, %)	14 (60.8)	2 (11.1)	0.005
Anteroseptal (n, %)	2 (8.7)	0	
Diffuse (n, %)	2 (8.7)	3 (16.7)	
Other (n, %)	4 (17.4)	8 (44.4)	
Original LLC			
Positive (n, %)	21 (91.3)	10 (55.6)	0.01
Updated LLC			
Positive (n, %)	22 (95.7)	13 (72.2)	0.07

*EGE* early gadolinium enhancement, *LGE* late gadolinium enhancement, *LLC* Lake Louise criteria, *LVEF* left ventricular ejection fraction, *LVEDVI* left ventricular end diastolic volume indexed to body surface area, *LVESVI* left ventricular end systolic volume indexed to body surface area, *RWMA* regional wall motion abnormality, *RVEF* right ventricular ejection fraction, *RVEDVI* right ventricular end diastolic volume indexed to body surface area, *RVESVI* right ventricular end systolic volume indexed to body surface area, *RWMA* regional wall motion abnormality

### T1 and T2 weighted imaging (Table 2)

**Fig. 1:**
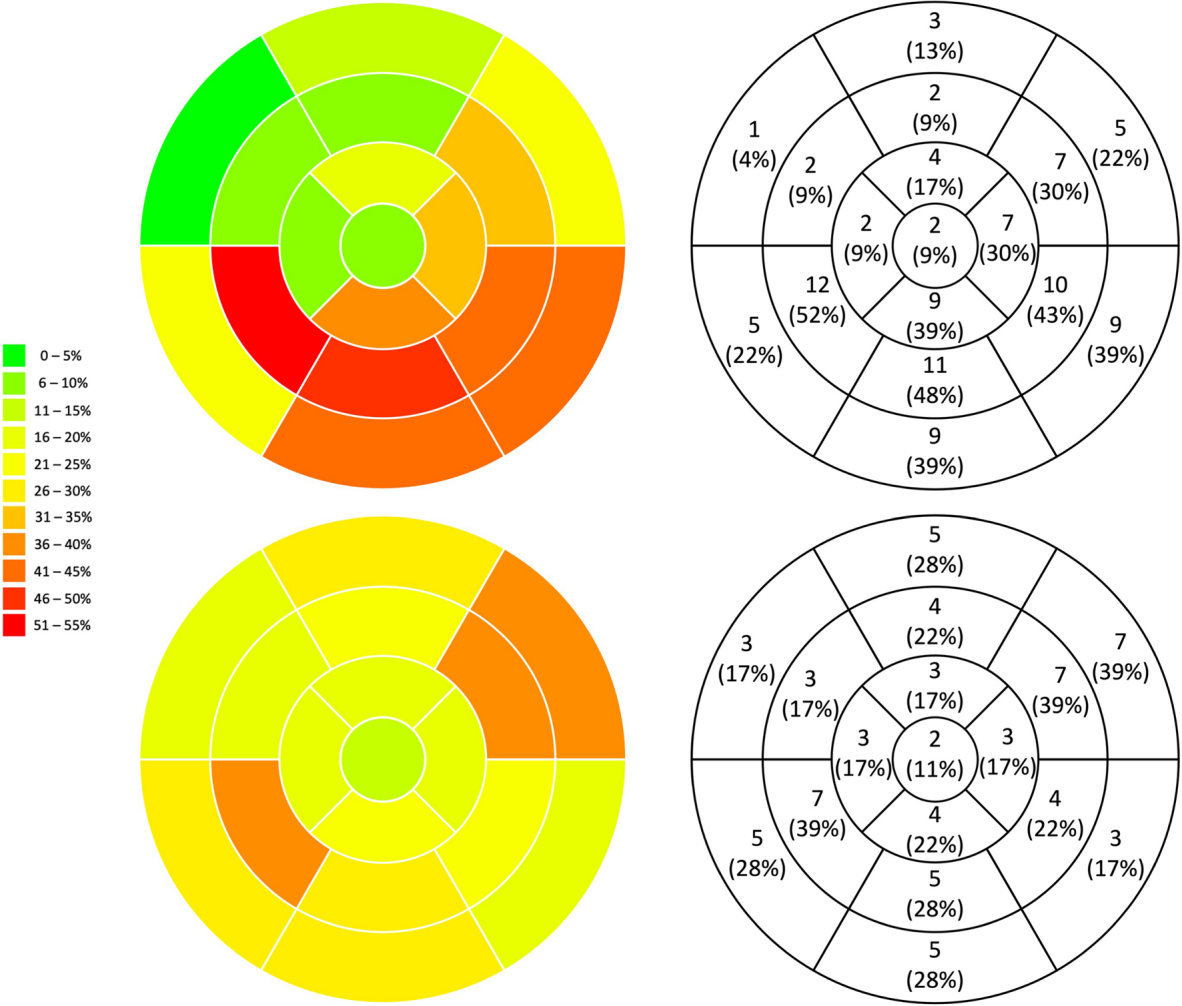
Comparison of the cumulative segments of late gadolinium enhancement in a 17-segment model of the left ventricle between patients with confirmed myocarditis presenting with infarct-like symptoms (top row) and heart failure symptoms (bottom row)

Patients with infarct-like presentation were significantly more likely to demonstrate oedema (91.3% vs. 61.1%, p = 0.03) and had significantly higher median total number of segments with oedema (3 vs. 1, p = 0.003) on STIR imaging. Although there was no statistical difference between the number of patients with LGE in the two groups (95.7% vs. 72.2, p = 0.07), those presenting with infarct-like symptoms had significantly higher median total number of segments with LGE (4.5 vs. 2.0, p = 0.02) (Fig. 1).

### LGE pattern

The distributions of LGE in both patients with infarct-like and HF presentation are summarised in Table 2 and illustrated in Fig. 1. The majority of patient presenting with infarct-like presentation had LGE localised to the inferolateral distribution (14 out of 22), compared to a patchy distribution in those with HF presentation (8 out of 13) (Additional file 1: Table S1).

### Parametric mapping

Parametric mapping only became available in our centre after the first five patients and was thus only performed in 36 patients. The findings are summarised and compared in Table 3. There was no significant difference in the number of patients with elevated native T1 relaxation time between the two groups. Patients with infarct-like presentation were significantly more likely to demonstrate elevated T2 relaxation.

**Table 3:** Findings of parametric mapping (n = 36)

	Infarct-like (n = 19)	Heart failure (n = 17)	p values
T1 relaxation time			
Mean (ms)	1096 ± 61	1081 ± 31	0.35
Elevated (n, %)	18 (94.7)	16 (94.1)	1.00
T2 relaxation time			
Median (ms)	52 (IQR 48–56)	48 (IQR 44–53)	0.12
Elevated (n, %)	13 (68.4)	6 (35.3)	0.04

### Lake Louise Criteria (LLC)

**Fig. 2:**
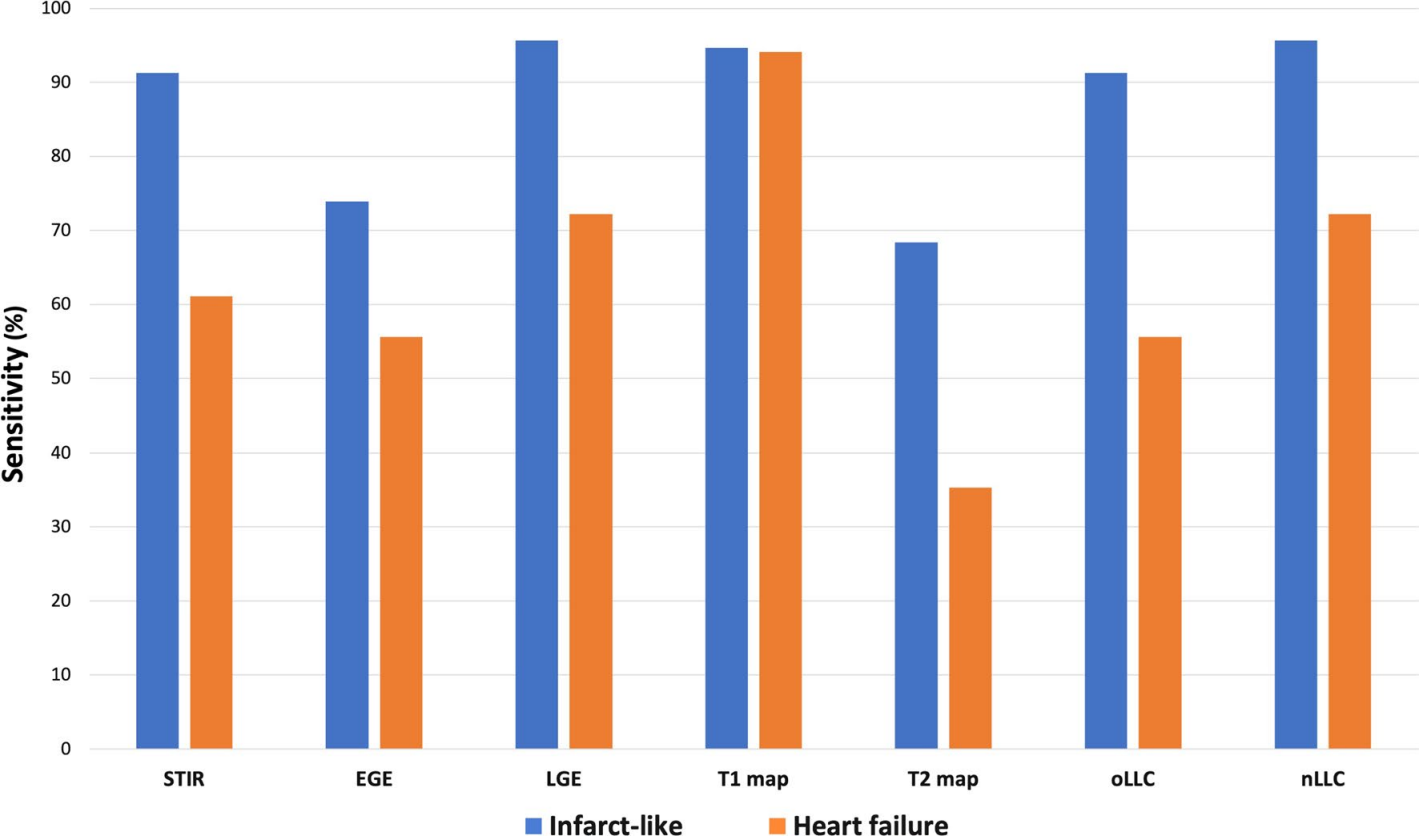
Comparison of the sensitivities of conventional cardiovascular magnetic resonance (CMR) sequences, parametric mapping and the original (oLLC) and new Lake Louise Criteria (nLLC)

Twenty-one (91.3%) patients in the infarct-like group fulfilled the original LLC which was significantly higher than ten (55.6%) patients in the HF group (p = 0.01). The updated LLC increased the diagnostic yield of the infarct-like group by one patient and the HF group by two patients, increasing the sensitivity to 95.7% and 72.2% respectively. The sensitivities of each individual criterion of the original and the updated LLC, along with those of both LLC are illustrated in Fig. 2.

## Discussion

The findings of this study demonstrated the contrasting patterns of CMR findings of patients with biopsy confirmed AM presenting with infarct-like and HF symptoms. Patients with infarct-like presentation had significantly higher LVEF and RVEF, fewer LV segments with wall motion abnormality, and a significantly higher number of LV segments demonstrating oedema and LGE. The sensitivity of both the original and updated LLC exceeded 90% in those with infarct-like presentation, while the sensitivity in the HF group improved from 55.6% with the original LLC to 72.2% with the updated LLC.

Myocardial oedema on T2 weighted imaging has previously been found to be more common on CMR of patients with AM and infarct-like presentation than those with HF presentation [10, 20]. It has been argued that this may reflect the contrasting time course in the disease process and could support the possible delay in presentation in patients with HF symptoms, as patients with acute onset chest pain are more likely to seek medical attention earlier [20]. Furthermore, viral infection of the myocytes in patients presenting with HF is thought to precede the onset of symptoms by several weeks with LV dysfunction resulting from diffuse myocardial damage caused by an autoimmune reaction to viral persistence [10, 11, 20, 21]. Thus by the time these patients present, progressive oedema reabsorption may have already occurred, which would then be poorly detectable on conventional imaging sequences [11, 20, 21]. In the current study, myocardial oedema was also both significantly more prevalent and present in more LV segments on CMR of patients with infarct-like presentation as compared to those presenting with HF. Despite the significant differences in both LV and RV systolic function between the two groups, the end diastolic dimensions of both ventricles on CMR were similar. The relatively preserved end diastolic ventricular dimensions in the presence of severely impaired systolic function in patients presenting with HF argues for acuteness of the pathology and potentially against a significant delay in presentation after the onset of illness. However, despite the lack of statistical significance of the LV end diastolic dimension between the two groups, the p value did approach 0.05. In view of the relatively small sample size of our study, the possibility of a type 2 statistical error has to be considered.

**Fig. 3:**
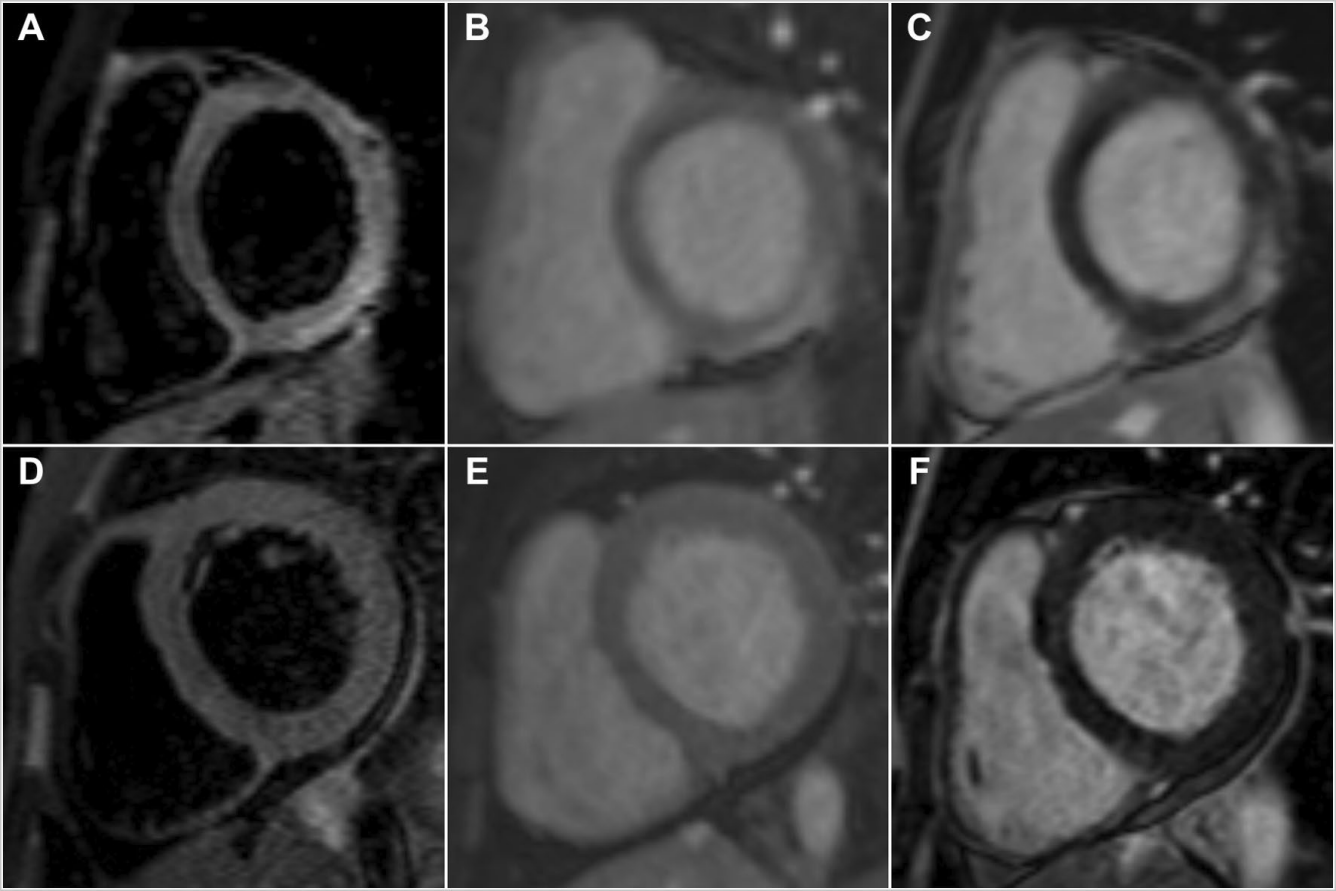
Contrasting patterns of CMR findings between patients with confirmed myocarditis presenting with infarct-like and heart failure symptoms. Top row: A 25-year-old male presenting with infarct-like symptoms and confirmed acute myocarditis on endomyocardial biopsy. **A** Short tau inversion recovery (STIR) imaging showing oedema in the inferolateral distribution. **B** Early gadolinium enhancement noted in the same distribution. **C** Bright subepicardial late gadolinium enhancement seen in the inferolateral wall. Bottom row: A 32-year-old male presenting with heart failure symptoms and confirmed acute myocarditis on endomyocardial biopsy. **A and B** No obvious oedema on STIR imaging nor early gadolinium enhancement. **C** Bland mid-myocardial late gadolinium enhancement seen in the septum

As a result of the low sensitivity of STIR imaging for myocardial oedema in patients with AM presenting with HF symptoms, the use of T1 weighted sequences detecting hyperaemia by EGE and necrosis or fibrosis by LGE are thought to be the preferred diagnostic approach in this group of patients [10]. The sensitivity of EGE in our study was the lowest of the three modalities of the original LLC in both group of patients, which supports the findings of previous studies demonstrating that its removal did not substantially hamper the diagnostic performance of the LLC [7, 22]. This is in contrast to LGE, which had the highest sensitivity amongst the three original LLC criteria in both groups. Although there was no significant difference between the number of patients with LGE in the two groups, those with infarct-like presentation had a significantly higher total number of LV segments with LGE when compared to the HF group. Furthermore, the pattern of LGE also appeared different between the two groups (Fig. 3), with the majority of the infarct-like group demonstrating bright subepicardial LGE in the inferolateral distribution compared to the predominant finding of patchy, mid-myocardial distribution of lower intensity in the heart failure group. The difference in the manifestation of LGE between the two groups has been ascribed to the contrasting underlying pathophysiological mechanisms affecting gadolinium kinetics [10, 11, 20–22]. Myocyte necrosis is thought to be the predominant form of cell death in patients with AM and infarct-like presentation [10, 11, 20, 21], whereas apoptosis predominates in those with HF presentation [8]. The fact that patients with infarct-like presentation had a mean hsTnT seven times higher than those presenting with HF in our cohort strongly supports this. Although previous authors have speculated that the different patterns of LGE observed in PVB19 and HHV6 myocarditis may be related to the differing tropism of these two viruses [10, 20, 21, 24], our findings are unique in that we were able to demonstrate two distinct patterns of LGE distribution based on clinical presentation alone regardless of the underlying viral pathogen.

The addition of parametric myocardial mapping techniques to the original LLC in 2018 was thought to significantly improve the diagnostic accuracy, especially in patients not presenting with infarct-like symptoms [7]. The quantification of native T1 and T2 relaxation times is thought to yield superior diagnostic performance as it allows for the detection of more subtle and more diffuse signal intensity abnormalities which might both otherwise be missed on conventional sequences [7, 11]. Native T1 relaxation times provide information from both intra- and extracellular space allowing for the detection of both myocardial oedema and fibrosis [25]. As a result, it may be elevated in both acute and chronic forms of myocarditis [26]. Nevertheless, it can provide superior diagnostic performance to T2 ratio and EGE and its specificity may exceed the original LLC when used in the appropriate clinical setting [27]. Similarly, we found T1 mapping to be highly sensitive in the diagnosis of AM and more interestingly, it was comparably excellent regardless of the manner of clinical presentation. However, the sensitivity of T2 mapping, a more specific marker of oedema and thus acute disease, was somewhat disappointing for both groups and significantly lower than those previously reported [5, 27]. Despite this, the application of the updated LLC increased the diagnostic yield of both groups of patients, supporting its utility especially in patients with non-infarct-like presentation as was previously found [11].

The findings of our study support the use of CMR as the first line investigation in patients with clinically suspected AM presenting with infarct-like symptoms, as the sensitivity of both the original and updated LLC exceeded 90% in this group. However, in patients with clinically suspected AM presenting with HF symptoms, EMB should be strongly considered early in the course of their presentation, as CMR may miss a significantly proportion of patients with AM in this group, and the yield of EMB is thought to be highest early in the course of the disease [1, 2, 4]. Furthermore, a definitive diagnosis in this group of patient may have significant bearing on future management and long-term prognosis [1, 2, 4].

### Limitations

This was a retrospective study performed in a single centre and its results may not be generalisable to other populations. However, the majority of our findings appeared to be similar to those previously reported in other studies. Although EMB confirmation of AM was considered the gold standard in this study and thus influenced the inclusion criteria, neither the specificity nor the sensitivity of EMB approach 100% and therefore cases with AM may have been missed. To mitigate this we studied only patients with biopsy confirmed AM. Unfortunately, this study design limited the ability to assess diagnostic specificity. The sample size of our study, although comparable with most studies recruiting patients with clinically suspected AM with EMB confirmation of AM, is relatively small. Extracellular volume (ECV) was not routinely measured in our cohort, however, with the sensitivity of native T1 mapping exceeding 90% in both groups, it is unlikely to have influenced our results significantly.

## Conclusion

The pattern of CMR findings and its diagnostic sensitivity differ in patients with AM presenting with infarct-like and HF symptoms. Although the sensitivity of the LLC improved with the addition of parametric mapping in the HF group, it remained lower than that of the infarct-like group, and suggests that EMB should be considered earlier in the course of patients with clinically suspected AM presenting with HF symptoms.

## Supplementary Information

**Additional file 1: Table S1. **Individual native T1 and T2 relaxation times of individual patients (n = 36).

## Data Availability

The datasets used and/or analysed during the current study are available from the corresponding author on reasonable request.

## Abbreviations

ACS: Acute coronary syndrome
AM: Acute myocarditis
AVB: Atrioventricular block
bSSFP: Balanced steady state free precession
CMR: Cardiovascular magnetic resonance
CMV: Cytomegalovirus
CRP: C-reactive protein
EBV: Epstein–Barr virus
ECG: Electrocardiogram
ECV: Extracellular volume fraction
EDD: End diastolic diameter
EGE: Early gadolinium enhancement
EMB: Endomyocardial biopsy
ESC: European Society of Cardiology
HF: Heart failure
HHV: Human herpesvirus
HIV: Human immunodeficiency virus
hs-cTnT: High sensitivity cardiac troponin T
IHC: Immunohistochemistry
ISFC: International Society and Federation of Cardiology
LGE: Late gadolinium enhancement
LLC: Lake Louise criteria
LV: Left ventricle/left ventricular
LVEF: Left ventricular ejection fraction
LVESV: Left ventricular end-systolic volume
NHLS: National Health Laboratory Services
PCR: Polymerase chain reaction
PVB19: Parvovirus B19
ROI: Region of interest
RV: Right ventricle/right ventricular
RVEF: Right ventricular ejection fraction
RVESV: Right ventricular end-systolic volume
RWMA: Regional wall motion abnormality
ShMOLLI: Shortened Modified Look-Locker inversion recovery
SIR: Signal intensity ratio
STIR: Short tau inversion recovery
TTE: Transthoracic echocardiography
VT: Ventricular tachycardia
WHO: World Health Organisation
