# Supplementary material for: Comparing the findings and diagnostic sensitivity of cardiovascular magnetic resonance in biopsy confirmed acute myocarditis with infarct-like vs. heart failure presentation
Source: J Cardiovasc Magn Reson. 2022 Dec 7;24:69. doi: 10.1186/s12968-022-00903-y (PMC9730564; doi:10.1186/s12968-022-00903-y)
Supplement: Supplementary file 1 — Additional file 1: Table S1. Individual native T1 and T2 relaxation times of individual patients (n = 36). [file 12968_2022_903_MOESM1_ESM.docx]

| **Table S1.** Individual native T1 and T2 relaxation times of individual patients (n = 36) | | |
| --- | --- | --- |
|  | T1 relaxation time (ms) | T2 relaxation time (ms) |
| Infarct-like (n = 19)  1  2  3  4  5  6  7  8  9  10  11  12  13  14  15  16  17  18  19 | \| 1090 \| \| --- \| \| 1180 \| \| 1071 \| \| 1075 \| \| 1056 \| \| 1139 \| \| 1111 \| \| 1052 \| \| 1063 \| \| 1128 \| \| 1059 \| \| 1069 \| \| 1251 \| \| 1183 \| \| 1070 \| \| 973 \| \| 1092 \| \| 1051 \| \| 1072 \| | \| 52 \| \| --- \| \| 61 \| \| 55 \| \| 53 \| \| 46 \| \| 52 \| \| 62 \| \| 48 \| \| 52 \| \| 57 \| \| 56 \| \| 55 \| \| 57 \| \| 48 \| \| 44 \| \| 47 \| \| 56 \| \| 48 \| \| 52 \| |
| Heart failure (n = 17)  1  2  3  4  5  6  7  8  9  10  11  12  13  14  15  16  17 | \| 1076 \| \| --- \| \| 1124 \| \| 1126 \| \| 1065 \| \| 997 \| \| 1099 \| \| 1083 \| \| 1096 \| \| 1072 \| \| 1077 \| \| 1055 \| \| 1061 \| \| 1088 \| \| 1102 \| \| 1052 \| \| 1112 \| \| 1092 \| | \| 63 \| \| --- \| \| 49 \| \| 70 \| \| 46 \| \| 44 \| \| 44 \| \| 49 \| \| 53 \| \| 56 \| \| 48 \| \| 45 \| \| 56 \| \| 47 \| \| 46 \| \| 47 \| \| 44 \| \| 52 \| |
